# Bullous striae distensae on Fitzpatrick scale V skin

**DOI:** 10.1016/j.jdcr.2023.08.032

**Published:** 2023-09-09

**Authors:** Laeshelle Basanoo, Narendra Persad, Akil Olliverrie, Rajeev P. Nagassar, Salma Mohammed, Jarred Brewster

**Affiliations:** a Intensive Care Unit, Anaesthetic Department, Sangre Grande Hospital, The Eastern Regional Health Authority, Sangre Grande, Trinidad and Tobago; b General Surgery Department, Sangre Grande Hospital, The Eastern Regional Health Authority, Sangre Grande, Trinidad and Tobago; c Post Graduate Year 2, Department of Internal Medicine, South Brooklyn Health, Trinidad and Tobago; d Department of Microbiology, Sangre Grande Hospital, The Eastern Regional Health Authority, Sangre Grande, Trinidad and Tobago

**Keywords:** bullae, bullous disorders, bullous striae distensae, Caribbean, Cushing’s syndrome, Fitzpatrick scale V, hypoalbuminemia, hypoproteinemia, melanated skin, skin of colour, stretch marks, striae distensae

## Introduction

Striae distensae, colloquially known as “stretch marks”, are prevalent in the adult human population.^1^, ^2^, ^3^, ^4^ Bullous striae distensae however, a complication of striae distensae, is exceedingly rare and sparsely encountered in medical literature.^5^, ^6^, ^7^, ^8^ It develops due to fluid accumulation within pre-existing striae and ameliorates upon resolution of the underlying etiological conditions.^5^^,^^7^

We report a case of bullous striae distensae, in a person with Fitzpatrick scale V skin, who had pre-existing striae distensae secondary to Cushing’s syndrome and morbid obesity. Marked hypoproteinemia, anasarca, and sepsis during her acute illness may have culminated in the development of this condition. We would like to highlight the rarity of this entity and illustrate its appearance in melanated persons of Caribbean origin, with Fitzpatrick scale V skin.

## Case presentation

A 38-year-old Caribbean woman, of mixed African and East Indian descent, was admitted to the intensive care unit for further management of septic shock, secondary to an abdominal incision infection, with associated wound dehiscence, following an incarcerated hernia repair. Medical history was significant for iatrogenic Cushing’s syndrome, class III obesity (body mass index 49), de novo type II diabetes mellitus and de novo hypertension, all secondary to a protracted course of oral prednisolone, at 40 mg orally once a day, for the duration of 3 years. This patient also reported a 1 year history of “stretch marks” most pronounced on the abdomen and flanks. On examination, she was diagnosed with bullous striae distensae as an incidental finding, in a similar distribution to her antecedent striae.

**Fig 1:**
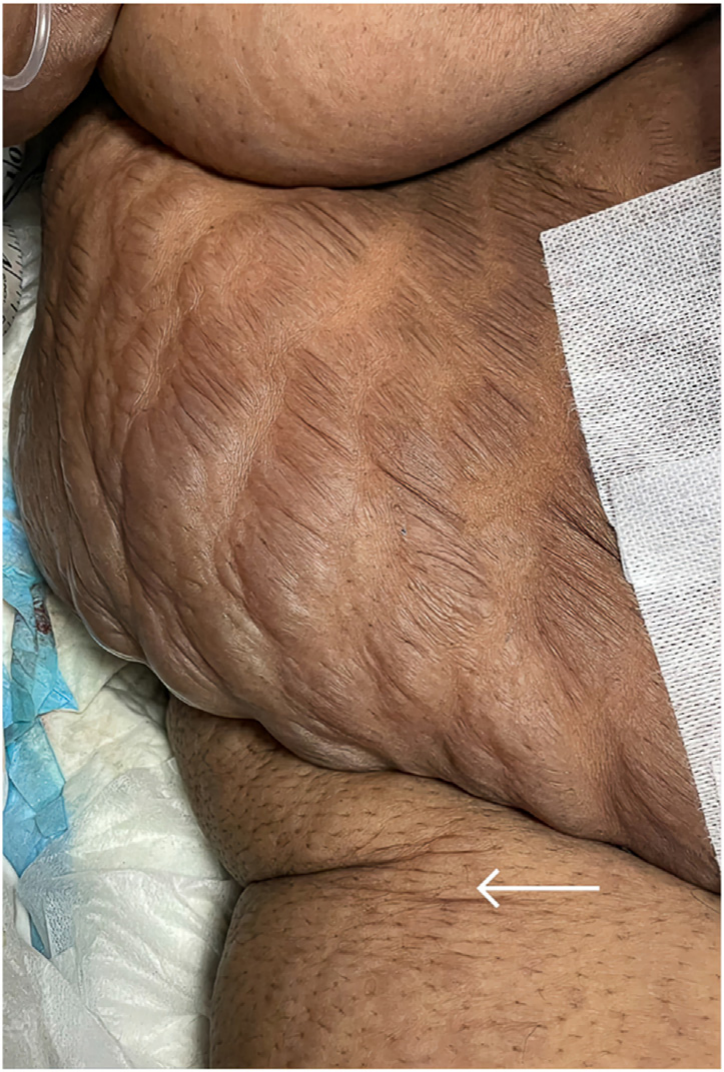
Photograph illustrating multiple fluid-filled bullous striae distensae in the distribution of her pre-existing striae of the *right* abdomen and flank, along with notable pedal edema (*whitearrow*), extending to the anterior superior iliac spine.

The patient described that prior to this admission, the striae were hyperpigmented linear streaks, flush with the surrounding unaffected skin. Subsequently, those located in gravity dependent regions progressed to become raised, distended, fluid filled and tense lesions, but remained nontender, nonpruritic, and nonerythematous (Fig 1). This dermatologic evolution was concurrent with her acute illness of severe hypoproteinemia and septic shock (see relevant laboratory investigations in Table I). In keeping with generalized anasarca, her physical examination was also positive for bilateral pedal edema extending to the anterior superior iliac spines (Fig 1) as well as the “moon face” and buffalo hump that is characteristic of Cushing’s syndrome.

**Table I:** Table showing patient’s laboratory investigations on admission and on discharge from the intensive care unit

Haematological investigations		
Parameter (range)	On admission	On discharge
Albumin (3.5-5.2 g/dL)	1.6	3.1
Total protein (6.4-8.3 g/dL)	3.8	5.9
AST (5-34 U/L)	234	89
ALT (0-55 U/L)	337	102
ALP (35-130 U/L)	96	47
GGT (9-64 U/L)	31	27
BUN (6-20 mg/dL)	15	18
Urea (16-48 mg/dL)	31.7	34.7
Creatinine (0.5-1.2 mg/dL)	0.4	0.9
Sodium (135-145 mmol/L)	137	139
Potassium (3.5-5.1 mmol/L)	4.12	4.8
White blood cell count	31.92	10.85
C- reactive protein (0.1-5 mg/dL)	127.36	39.2
Erythrocyte sedimentation rate (0-30 mm/hr)	66	N/A
Urine dipstick	3+ protein	1+ protein

Aspirate investigations		
	LDH (range: 135-225U/L)	Total protein (mg/dL)
Blood results	350	4.7
Aspirate results	121	0.8
Ratio	0.34	0.17

*ALP*, Alkaline phosphatase; *ALT*, alanine transaminase; *AST*, aspartate aminotransferase; *BUN*, blood urea nitrogen; *GGT*, gamma glutamyl transferase; *LDH*, lactate dehydrogenase.

**Fig 2:**
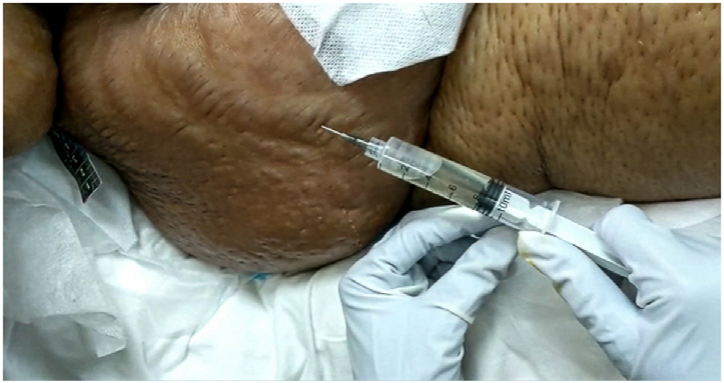
Photograph demonstrating aspiration of serous, straw colored fluid from a tense bullous striae on the right flank, for further evaluation.

**Fig 3:**
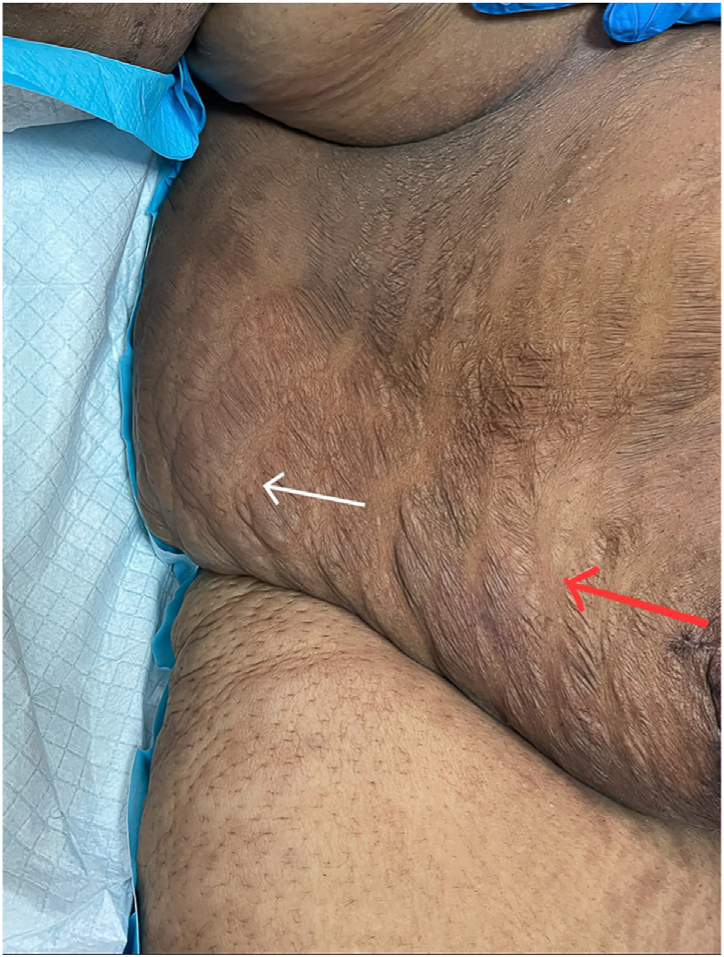
Photograph illustrating both persistence (*whitearrow*) and partial resolution (*red arrow*) of the bullous striae distensae.

**Fig 4:**
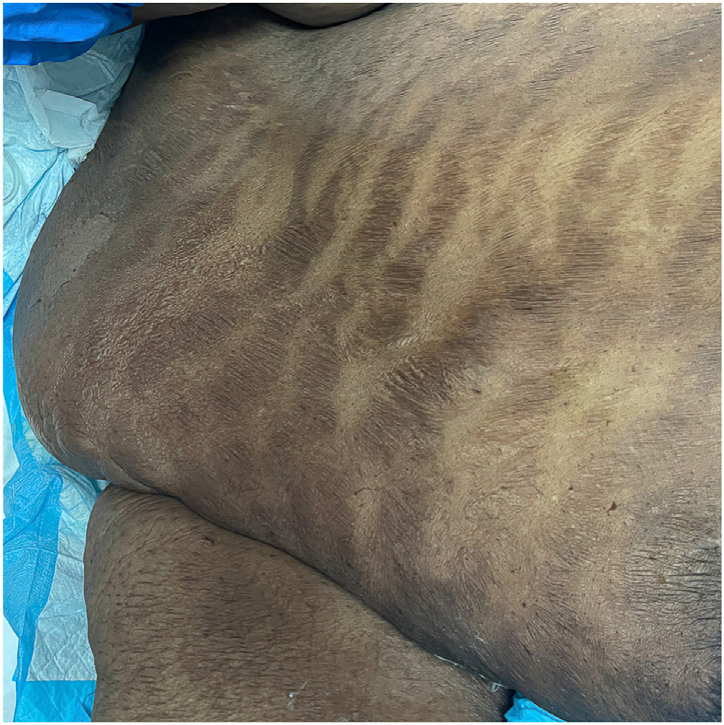
Photograph of *right* flank illustrating complete resolution of the bullous striae distensae.

Aspiration of bullae on the abdominal wall for further investigation, revealed clear, straw colored fluid (Fig 2), which was confirmed to be a sterile transudate (see relevant laboratory investigations in Table I). As the patient’s clinical condition improved—namely resolution of sepsis and normalizing of both the albumin and total protein levels—the bullous striae distensae gradually resolved without dermatologic intervention (Figs 3 and 4).

## Discussion

Bullous striae distensae occurs due to fluid transudation into large, pre-existing striae.^5^ Its disease etiology is multifactorial and has documented associations with nephrotic syndrome, systemic lupus erythematosus, and hepatic dysfunction.^3^^,^^5^, ^6^, ^7^, ^8^ The unifying, cornerstone feature is decreased oncotic pressure, secondary to hypoalbuminemia,^3^^,^^5^, ^6^, ^7^, ^8^ which favors the third spacing of fluid. In this patient, compounding factors that promoted anasarca and fluid shifts into the interstitium were decreased oncotic pressure and sepsis.

Albumin is a large, negatively charged protein, and as such, is able to attract water molecules to itself, exerting a high oncotic pressure and retaining fluid intravascularly.^9^ Hepatic impairment, poor nutritional status, and increased albumin catabolism, due to acute illness, significantly reduced the circulating intravascular albumin in our patient, resulting in loss of fluid to the interstitium.^9^ Additionally, sepsis provokes the release of inflammatory cytokines which increase vascular permeability, further exacerbating fluid leakage. Despite developing generalized edema, the striking observation of this case was the significant fluid accumulation within the striae.

The skin of striae distensae is inherently weaker than normal skin, but its precise pathophysiology is not fully established.^1^^,^^2^^,^^4^ The initial insult is mechanical stress on the skin, such as stretching during rapid weight gain, resulting in dermal tears, activation of an inflammatory response and distortion of the extracellular matrix.^2^^,^^4^ This remodeling results in scarred skin containing less elastin, collagen and fibrillin, with reduced tensile strength.^1^^,^^2^^,^^4^ This reduction in tensile strength, in combination with higher glycosaminoglycan content, precipitated the preferential distention and fluid accumulation within the striae, when compared to the adjacent normal skin, resulting in the bullous striae distensae.^2^

Interestingly, the majority of patients in literature who developed bullous striae distensae, were receiving some form of systemic corticosteroid,^3^^,^^5^^,^^7^^,^^8^ including ours. Systemic corticosteroids have an established link to atrophic skin changes that precipitate the violaceous striae of hypercortisolism. The clinical indication for use is usually for their anti-inflammatory properties, however these same attributes impair wound healing,^10^ as noted in our patient with abdominal incision wound dehiscence, suppress keratinocyte proliferation, and reduce collagen synthesis.^4^^,^^10^

Despite its dramatic appearance, bullous striae distensae is generally a benign condition,^3^^,^^7^ although the potential for rupture or infection is recognized.^3^^,^^5^ Management should be tailored to rectifying the etiology of the low oncotic state and other exacerbating factors.^3^^,^^5^

This patient’s septic shock was managed with vasopressor support (noradrenaline infusion), physical debridement of the infected tissue, and culture directed intravenous antibiotics (imipenem 500 mg intravenously twice a day.) Improvement in the hypoalbuminemia was achieved via high protein enteral feeds over the course of several weeks and eliminating hepatotoxic drugs from her regimen. This combination of supportive management resulted in the complete resolution of the bullous lesions, without specific dermatological intervention.

We would like to highlight the rarity of bullous striae distensae and contribute to the literature by showing its appearance in persons with melanated skin, of Caribbean ancestry.

## Conflicts of interest

None disclosed.
